# A Sensitive and Accurate Electrochemical Sensor Based on Biomass-Derived Porous Carbon for the Detection of Ascorbic Acid

**DOI:** 10.3390/molecules30142980

**Published:** 2025-07-15

**Authors:** Yashuang Hei, Lisi Ba, Xingwei Shi, Huanhuan Guo, Sisi Wen, Bingxiao Zheng, Wenjie Gu, Zhiju Zhao

**Affiliations:** 1 Functional Polymer Materials Research and Development and Engineering Application Technology Innovation Center of Hebei Province, Xingtai University, Xingtai 054001, China; balisi814@163.com (L.B.); claireguo1124@hotmail.com (H.G.); 18844115900@163.com (S.W.); 15116903609@163.com (B.Z.); 2 Xingtai Key Laboratory of Biomimetic and Catalytic Materials, Xingtai 050041, China; shixingwei0213@163.com; 3 College of Chemical Engineering and Biotechnology, Xingtai University, Xingtai 054001, China; 15028335517@163.com

**Keywords:** ascorbic acid, biomass, electrochemical sensor, porous carbon

## Abstract

Ascorbic acid (AA) is a vital biomarker for human metabolic processes, and many diseases are strongly linked to aberrant variations in its content. It is crucial to detect the levels of AA with sensitivity, speed, and accuracy. In this work, three-dimensional honeycomb-like porous carbons derived from discarded walnut (green) husks (DWGH-HCPCs) were synthesized using a process involving hydrothermal treatment, freeze-drying, and carbonization. The DWGH-HCPCs, with a high specific surface area of 419.72 m^2^ g^−1^, large pore volume of 0.35 cm^3^ g^−1^ and high density of defective sites, are used to fabricate the electrochemical sensor for the detection of AA. The electrochemical performance of the DWGH-HCPC-modified glassy carbon electrode (GCE) (DWGH-HCPC/GCE) was investigated through chronoamperometry, differential pulse voltammetry, and cyclic voltammetry. Compared with the GCE, the DWGH-HCPC/GCE exhibits higher sensitivities (34.7 μA mM^−1^ and 22.7 μA mM^−1^), a wider linear range (10–1040 μM and 1040–3380 μM), and a lower detection limit (0.26 μM) for AA detection. Specifically, the real sample concentrations of AA in beverages and artificial urine were successfully identified by DWGH-HCPC/GCE. Additionally, the DWGH-HCPC/GCE demonstrated great feasibility in the simultaneous detection of AA, dopamine (DA), and uric acid (UA). Therefore, as a green, eco-friendly, and low-cost electrode modifier, DWGH-HCPCs have broad prospects in the development of electrochemical sensing platforms for food and medical applications.

## 1. Introduction

L-ascorbic acid (AA), commonly referred to as vitamin C, is a crucial water-soluble biomolecule with significant antioxidant properties that can be obtained from fresh fruits, green vegetables, and various dietary supplements [1,2,3]. Although AA cannot be synthesized endogenously in the human body, it plays a vital role in human metabolism due to its antioxidant properties and ability to combat bacterial infections, thereby protecting the body from oxidative stress and free radical-induced diseases such as cancer, liver and kidney diseases, or mental disorders [4,5,6,7]. According to established guidelines, the recommended daily intake of AA for healthy adults is approximately 90 mg per day [8]. While excessive ascorbic acid (AA) consumption may cause gastrointestinal disorders, elevated female infertility risk, and embryonic developmental impairment, severe deficiency leads to scurvy and is associated with an increased incidence of cardiovascular diseases, Parkinson’s disease, and rheumatoid arthritis [9,10,11]. Additionally, AA is incorporated into select food and beverage products as a functional antioxidant additive to enhance oxidative stability and inhibit organoleptic deterioration, particularly color and flavor changes [12]. Given these considerations, accurate quantification of AA concentrations in both food matrices and biological fluids is critical for ensuring dietary quality, monitoring nutritional status, and assessing potential health risks.

Multiple analytical techniques have been employed for the determination of AA, including fluorescence analyses [13], high-performance liquid chromatography [14], capillary electrophoresis [15], the spectrophotometric method [16], electrochemical methods [17], and gas chromatography [18]. Nonetheless, these conventional techniques are fundamentally constrained by multiple inherent limitations—including bulky instrumentation, significant operator expertise, elevated operational costs, complex procedural workflows, stringent laboratory requirements, and prolonged analysis time—rendering them unsuitable for highly sensitive online monitoring applications [19,20]. In contrast, electrochemical methods exhibit superior analytical performance characteristics, such as real-time detection and analysis, high sensitivity, fast reaction time, and good selectivity, which can be applied broadly in pollutant monitoring, medical diagnosis and biochemical sensing [17,21]. Although AA can be directly oxidized on bare electrodes, its electrochemical detection in complex matrices is often compromised by interference from coexisting electroactive species (e.g., dopamine (DA) and uric acid (UA)) due to overlapping oxidation potentials, leading to poor reproducibility and suboptimal detection performance with conventional unmodified electrodes [22]. Therefore, modification of electrodes is preferable for electrochemical detection of AA. Substantial research efforts have recently focused on engineering advanced electrode modification materials, encompassing metal nanoparticles, MOF-based materials, artificial enzymes and porous carbon (PC) materials, among others [23,24,25,26].

PCs have attracted significant research attention as electrode modifiers due to their exceptional physicochemical properties, including large specific surface area, abundant active sites, short diffusion pathways for mass transfer, and accessible chemical chemistry, which enable their successful widespread applications in electrochemical sensors. Lee et al. developed a highly sensitive electrochemical sensor based on hierarchical PCs with sponge-like edge structures for the detection of Cd(Ⅱ) and Pb(Ⅱ) with low detection limits (LOD) of 0.41 and 0.7 μg L^−1^, respectively [27]. Zhao et al. successfully proposed a yolk–shell hierarchically PC (N-CF@N,P-CF) for the sensing of hydroquinone, catechol, acetaminophen, dopamine and uric acid with low LODs of 15.4 nM, 18.8 nM, 16.2 nM, 22.2 nM, and 24.5 nM, respectively [28]. A functionalized hybridized nanoporous carbon (H-NPC)-encapsulated flexible 3D porous graphene-based epidermal patch was fabricated for monitoring sweat glucose and lactate with high sensitivities of 82.7 μA mM^−1^ cm^−2^ and 204 nA mM^−1^ cm^−2^, respectively [29]. The high production costs and intricate synthesis protocols currently limit the scalable manufacturing and real-world deployment of PCs. The utilization of biomass precursors for PC synthesis has recently attracted considerable attention, driven by the dual advantages of economic viability and environmental compatibility [30,31]. Biomass-based PCs have also been applied in many fields, including electrochemical sensors [32,33].

**Scheme 1:**
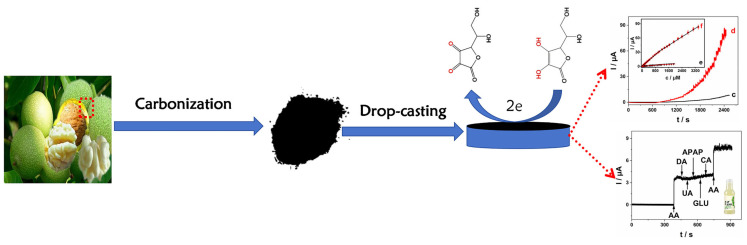
Synthesis of DWGS-HCPCs for the amperometric determination of AA.

In this work, a sensitive and accurate electrochemical sensor based on three-dimensional honeycomb-like porous carbons derived from discarded walnut (green) husks (DWGH-HCPCs) is fabricated for the detection of AA (Scheme 1). The DWGH-HCPCs are fabricated via hydrothermal treatment, freeze-drying, and high-temperature carbonization, exhibiting a large specific surface area with high-density active sites on rough surfaces and short diffusion pathways for mass transfer, which are attributed to the porous structure and which synergistically enhance the electrochemical performance of a DWGH-HCPC-based electrochemical sensor. The DWGH-HCPC-based electrochemical sensor exhibits a wide linear range, a low detection limit, fabrication simplicity (e.g., drop-casting), cost-effectiveness (selecting almost cost-free biomass waste), and the potential for simultaneous detection of multiple substances.

## 2. Results and Discussion

### 2.1. Characterization of DWGS-HCPCs

**Figure 1:**
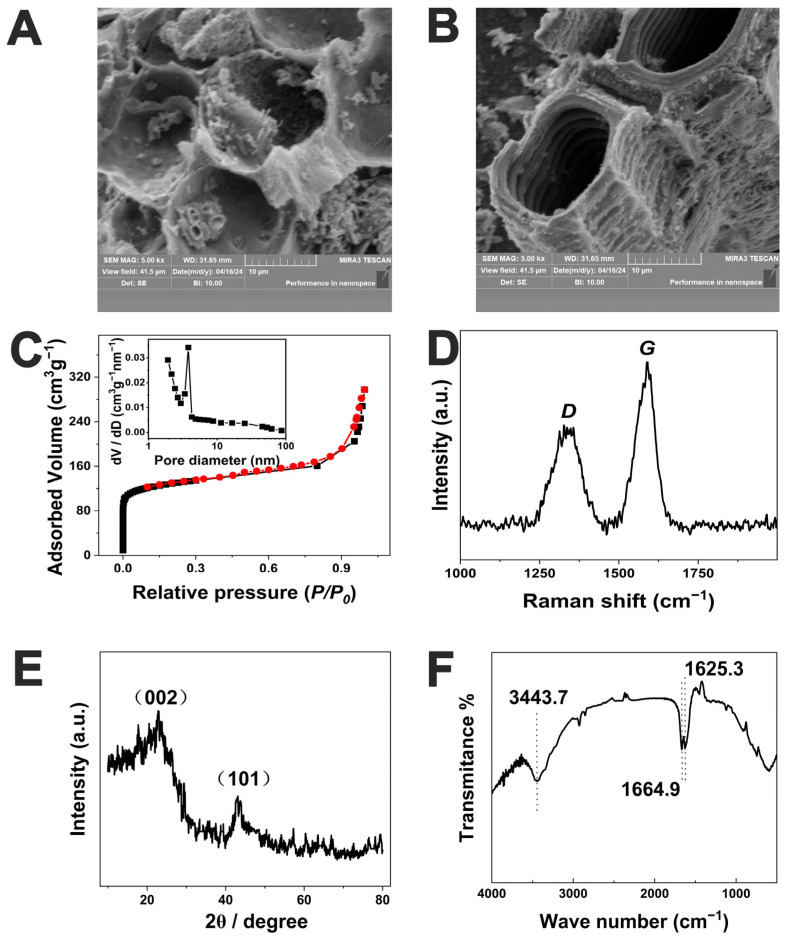
(**A**,**B**) SEM images of DWGS-HCPCs. (**C**) N_2_ adsorption–desorption isotherm of DWGS-HCPCs. Inset: the pore size distribution of DWGS-HCPCs. (**D**) Raman spectrum of DWGS-HCPCs. (**E**) XRD pattern of DWGS-HCPCs. (**F**) FT-IR spectrum of DWGS-HCPCs.

The surface morphology and microstructure of DWGS-HCPCs were systematically characterized using scanning electron microscopy (SEM). As depicted in Figure 1A,B, DWGS-HCPCs exhibit a honeycomb-like porous architecture, which arises from thermally induced structural reorganization. As the carbonization temperature increases, the carbon skeleton undergoes tensile deformation, resulting in hierarchical expansion and stratification of pore channels that form multi-layered annular configurations. This structural evolution leads to a large specific surface area, surface roughness with well-defined pore channel delineation, and exposure of abundant active functional groups on the carbon matrix surface [34]. Figure 1C further displays the porosity characteristics through nitrogen (N_2_) adsorption-desorption isotherm. According to the Brunauer-Emmett-Teller (BET) analysis, the specific surface area of a DWGS-HCPC was found to be 419.72 m^2^ g^−1^. The pore volume (0.32 cm^3^ g^−1^) and the pore size distribution, mainly centered at about 3.77 nm, were obtained in the inset of Figure 1C via Barrett-Joyner-Halenda (BJH) analysis. The hierarchical porous architecture and high specific surface area of DWGS-HCPCs collectively promote shortened ion diffusion pathways and rapid electron transport kinetics, thereby enhancing electrochemical reaction efficiency.

The Raman spectrum of the DWGS-HCPC in Figure 1D displays two prominent characteristic peaks. The D-band centered at approximately 1335 cm^−1^ is attributed to structural defects and disorder in carbon materials, primarily associated with sp^3^-hybridized carbon atoms [35]. The G-band at 1590 cm^−1^ corresponds to the sp^2^-hybridized graphitic domains, reflecting the degree of graphitization [35]. The intensity ratio of the D-band to G-band (I_D_/I_G_) for DWGS-HCPCs is calculated to be 1.64, which is higher than those ratios of N, P-doped porous carbon materials (HP-COF-C-600, 1.24) [36], porous carbon in durian shell doped with heteroatoms (S-DCK-3, 1.77) [37], and Co_6.8_Se_8_ embedded in porous carbon (Co_6.8_Se_8_@NPC, 1.07) [38], in turn indicating the presence of disorder structure or a significant amount of defects in DWGS-HCPCs, which is favorable for electrochemical reactions. X-ray diffraction (XRD) analysis further elucidates the structural features of DWGS-HCPCs (Figure 1E). The broad diffraction peaks at 22.9° and 43.7° are assigned to the (002) plane and the (101) plane of graphitic carbon [39]. The information of the surface functional groups of DWGS-HCPCs was studied through the Fourier transform infrared (FT-IR) spectroscopy shown in Figure 1F. The band at around 3443.7 cm^−1^ is attributed to the O-H stretching vibrations. The bands at 1664.9 cm^−1^ and 1625.3 cm^−1^ correspond to the C=O stretching vibrations in aromatic carboxylic acids and amides, respectively. The oxygen-containing groups (e.g., -OH) can improve the hydrophilicity and the electrochemical performance of DWGS-HCPCs.

### 2.2. Electrochemical Evaluation of DWGS-HCPC/GCE

**Figure 2:**
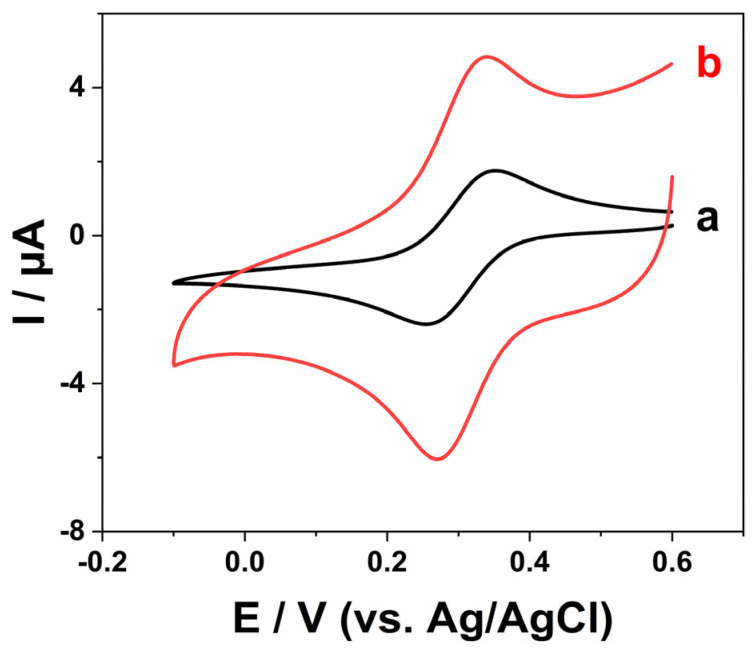
CVs at the GCE (a) and DWGS-HCPC/GCE (b) in 5 mM K_3_[Fe(CN)_6_] containing 0.1 M KCl. Scan rate: 10 mV s^−1^.

The electrochemical behavior of DWGS-HCPC/GCE is first evaluated by cyclic voltammetry in 5 mM K_3_[Fe(CN)_6_] containing 0.1 M KCl. The effect of different modification volumes (2 μL, 4 μL, 6 μL, 8 μL, 10 μL) on the electrochemical performance of the electrode was evaluated. The 8 µL modification volume exhibited the smallest peak-to-peak potential separation (∆E_p_) and the highest current response. Therefore, the 8 µL modification volume was selected for subsequent experiments. In Figure 2, the modification of 8 μL of DWGS-HCPC/GCE (a, 30.72 μA) exhibits a significantly reduced ∆E_p_ of 67 mV and a 1.39-fold increase in the peak current of the reduction reaction compared with the bare GCE (b, ∆E_p_ = 93 mV, 22.16 μA), indicating enhanced electrochemical reversibility due to improved electron transfer kinetics at the modified interface [40]. According to the Nicholson method [41], the heterogeneous electron transfer constant (*k^0^*) of ${Fe(CN)}_{6}^{4-}$/${Fe(CN)}_{6}^{3-}$ at DWGS-HCPC/GCE and GCE are 0.00987 and 0.00207 cm s^−1^, respectively. Compared with the GCE, the higher *k^0^* value of DWGS-HCPC/GCE shows that the presence of DWGS-HCPCs accelerates the rate of electron transport. Furthermore, the Randles-Sevcik equation [42] indicates that the electroactive surface area of the DWGS-HCPC/GCE (0.088 cm^2^) is higher than that of the GCE (0.064 cm^2^), which is the primary cause of the increased background current measured on the DWGS-HCPC/GCE when compared with the GCE.

The electrochemical impedance spectroscopy (EIS) method was used to investigate the ability of electron transfer for GCE and DWGS-HCPC/GCE. In Figure S1, the EIS plots display a semicircular section at high frequency and a linear section at low frequency. The charge transfer resistance (*R*ct) was reported to correspond to the diameter of the semicircle. The DWGS-HCPC/GCE exhibits a smaller *R*ct (2.86 Ω) than that at GCE (100.2 Ω), which indicates that the existence of DWGS-HCPCs can accelerate the rate of electron transfer.

### 2.3. Electrochemical Catalysis and Detection of Ascorbic Acid (AA) at DWGS-HCPC/GCE

**Figure 3:**
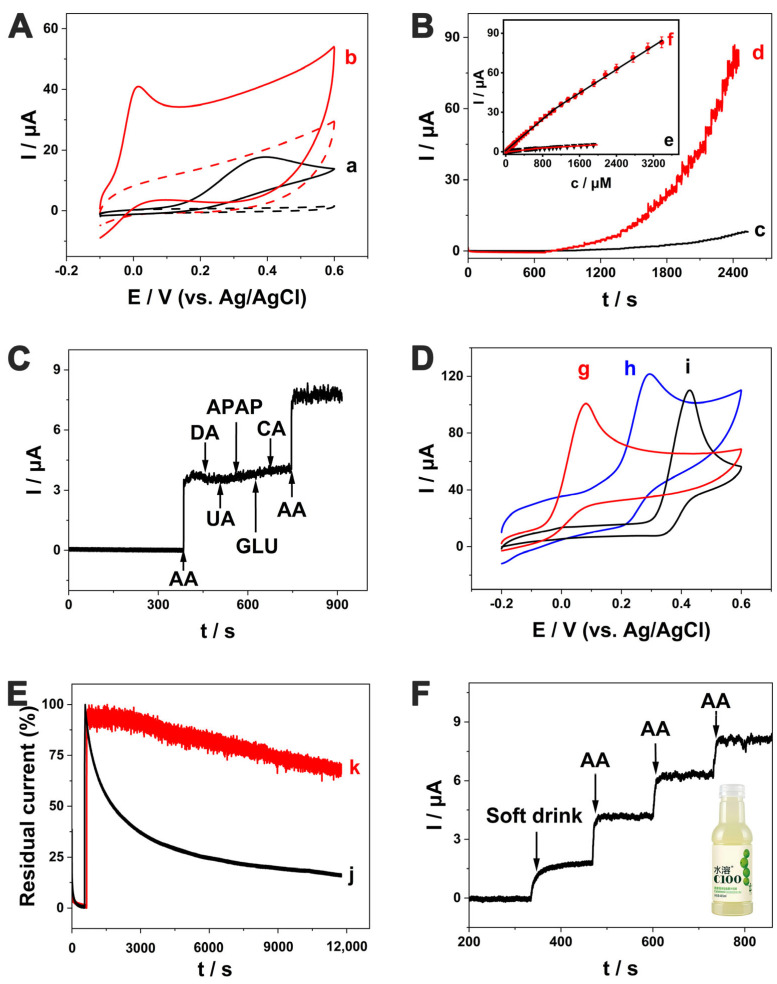
(**A**) CVs at the GCE (a) and DWGS-HCPC/GCE (b) for 3 mM AA. Dotted lines: background responses. Scan rate: 10 mV s^−1^. Electrolyte: N_2_-saturated 0.1 M pH 7.0 PBS. (**B**) Current vs. time curves at the GCE (c) and DWGS-HCPC/GCE (d) with continuous increase of the amount of AA. Inset: corresponding calibration curves of AA at the GCE (e) and DWGS-HCPC/GCE (f). (**C**) Current vs. time curve at the DWGS-HCPC/GCE with the addition of 100 μM AA, 150 μM DA, 100 μM UA, 100 μM CA and 1 mM GLU. (**D**) CVs for 6 mM AA (g), 3 mM DA (h) and 6 mM UA (i). Electrolyte: N_2_-saturated 0.1 M pH 7.0 PBS. Scan rate: 10 mV s^−1^. (**E**) The current vs. time curves with 300 μM AA at the GCE (j) and DWGS-HCPC/GCE (k). (**F**) The current vs. time curves with the real sample and three equal amounts of standard AA solutions (100 μM, 100 μM, 100 μM) at DWGS-HCPC/GCE. Applied potential in (**B**,**C**,**E**,**F**): 0.015 V. Electrolyte in (**B**,**C**,**E**,**F**): vigorously magnetically stirred N_2_-saturated 0.1 M pH 7.0 PBS.

The cyclic voltammograms (CVs) GCE (a) and DWGS-HCPC/GCE (b) in the absence and presence of 3 mM AA are shown in Figure 3A. A slow electron transfer kinetic at GCE is demonstrated by the irreversible AA electro-oxidation, which is accompanied by an oxidation peak potential of 0.396 V. At DWGS-HCPC/GCE (0.015 V), the oxidation peak potentials of AA are negatively displaced by 381 mV in comparison with GCE. In addition, the oxidation peak current for AA at DWGS-HCPC/GCE exhibits a 2.25-fold enhancement compared with the bare GCE. The presence of DWGS-HCPCs, which greatly facilitated electron transfer, may be the cause of the low oxidation overpotential of AA at DWGS-HCPC/GCE [43].

Based on the superior electrochemical performance for AA electrooxidation, amperometry is used to assess the sensing application of DWGS-HCPC/GCE to AA at the applied potential of 0.015 V (e.g., the oxidation peak potential of AA in CV). The current–time curves at GCE (c) and DWGS-HCPC/GCE (d) are shown in Figure 3B using varying AA concentrations in 0.1 M vigorously magnetically stirred N_2_-saturates phosphate buffered solution (PBS) (pH = 7.0). DWGS-HCPC/GCE demonstrated a quick current response (less than 5 s) and a clear current increase to the addition of AA, whereas GCE showed a significantly lesser response to AA. The inset of Figure 3B displays the calibration curves for AA detection at GCE (e) and DWGS-HCPC/GCE (f). The current response shows a linear dependence on AA concentration with three consecutive intervals of 10–1040 μΜ and 1040–3380 μΜ, demonstrating the capability of the DWGS-HCPC/GCE for multiscale quantitative analysis. The DWGS-HCPC/GCE also demonstrates high sensitivities (34.7 μA mΜ^−1^ and 22.7 μA mΜ^−1^) and low detection limit (LOD, 0.26 μM). The DWGS-HCPC/GCE exhibits analytical performance measures, such as linear range and LOD, that are comparable to or better than those of recently published electrochemical AA sensors [44,45,46,47,48,49], as shown in Table 1. Furthermore, the performance of the electrochemical detection of AA on DWGS-HCPC/GCE was compared with other classical methods for detecting AA, as shown in Table S1 [13,14,15,16,50].

In the investigation for the selectivity of the AA electrochemical sensor, uric acid (UA), acetaminophenol (APAP), dopamine (DA), glucose (GLU) and citric acid (CA) were employed as the possible interfering species for AA detection. In Figure 3C, the amperometric current responses of AA and the possible interferences at DWGS-HCPC/GCE were investigated at 0.015 V. The negligible signals generated by the interferences were found by the interferences on DWGS-HCPC/GCE, demonstrating the good selectivity and potential for detecting complex real samples. Figure 3D shows that the peak potential for AA electrooxidation (0.081 V, g) is lower than the onset potential for UA (0.285 V, i) and is comparable to that of DA (h). There are almost no electrochemical oxidations of DA and UA at DWGS-HCPC/GCE at the potential of 0.081 V, hence there are almost no interferences from DA and UA for AA detection at DWGS-HCPC/GCE with the applied potential of 0.081 V.

The electrode surface is susceptible to adsorption by the oxidation products of small biomolecules during detection. To evaluate the anti-fouling performance of DWGS-HCPC/GCE during AA detection, its operational stability was determined by measuring the current–time response of 300 μΜ AA. The current response at DWGS-HCPC/GCE (k), as illustrated in Figure 3E, still holds 75.62% of the original value, which is higher than 26.09% at GCE (j), suggesting a greater antifouling capacity at DWGS-HCPC/GCE. Furthermore, the storage stability of the DWGS-HCPC/GCE (stored in a 4 °C refrigerator) was further investigated. The performance of the DWGS-HCPC/GCE for 300 μΜ AA was studied on the 1st day, the 3rd day, the 5th day and the 7th day respectively. In Figure S2, the results show that the DWGS-HCPC/GCE was relatively stable.

Six identical DWGS-HCPC/GCEs were prepared and tested using the chronoamperometry in 0.1 M pH = 7.0 PBS containing 100 μΜ AA. In Figure S3, the results show that the electrochemical responses of the six electrodes were almost the same, proving that the DWGS-HCPC/GCE shows good reproducibility.

### 2.4. Real Samples

DWGS-HCPC/GCE has a tremendous potential for AA detection in real samples due to its outstanding performance for AA detection. Therefore, the amounts of AA in a commercial beverage (namely water-soluble C100) were evaluated using the standard addition method at DWGS-HCPC/GCE, shown in Figure 3F. The mean recoveries for water-soluble C100 range from 93% to 103%. The AA concentration in water-soluble C100, measured by DWGS-HCPC/GCE, is calculated to be 1.26 mM, which is close to the label value (1.28 mM) provided by the company, demonstrating the enormous potential of DWGS-HCPC/GCE for accurate and reliable AA analysis in a variety of domains ranging from the pharmaceutical industry to food analysis.

### 2.5. Electrochemical Behavior of the Coexistence of AA, DA and UA at DWGS-HCPC/GCE

**Figure 4:**
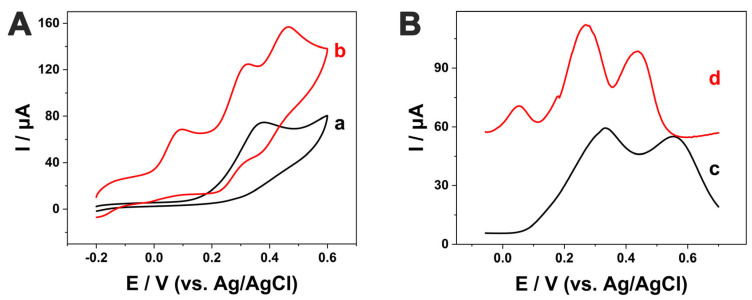
(**A**) CVs at the GCE (a) and DWGS-HCPC/GCE (b) for 6 mM AA, 3 mM DA and 6 mM UA. Scan rate: 10 mV s^−1^. (**B**) DPVs at the GCE (c) and DWGS-HCPC/GCE (d) for 6 mM AA, 3 mM DA and 6 mM UA. Electrolyte in A and B: N_2_-saturated 0.1 M pH 7.0 PBS.

Based on the previous results of the detection of AA at DWGS-HCPC/GCE, the potential of DWGS-HCPC/GCE for the simultaneous detection of AA, DA and UA was examined by cyclic voltammetry and differential pulse voltammetry. The CVs of 6 mM AA, 3 mM DA and 6 mM UA in PBS (0.1 M, pH = 7.0) at DWGS-HCPC/GCE (b) and GCE (a) are displayed in Figure 4A. The GCE only displays one anodic peak at 0.381 V, suggesting that the GCE is unable to detect AA, DA and UA simultaneously. On the contrary, three distinct anodic current peaks of AA (0.097 V), DA (0.322 V) and UA (0.465 V) can be identified. The oxidation peak separations between AA and DA, DA and UA, UA and AA are 225 mV, 143 mV and 368 mV, respectively, which affirms the capacity of DWGS-HCPC/GCE to simultaneously and sensitively detect AA, DA and UA using cyclic voltammetry. Moreover, the simultaneous detection of AA, DA and UA is accomplished using differential pulse voltammetry. Figure 4B shows the DPVs of 6 mM AA, 3 mM DA and 6 mM UA in PBS (0.1 M, pH = 7.0) at DWGS-HCPC/GCE (d) and GCE (c). The electrooxidation potentials of the three biomolecules at GCE overlapped and produced two broad peaks, suggesting that the GCE is unable to discriminate between AA, DA and UA. Nonetheless, a high resolution is demonstrated by the oxidation peaks of AA, DA and UA at the DWGS-HCPC/GCE, which are detected at 0.052 V, 0.268 V and 0.438 V, respectively. The significant difference in the oxidation peak potentials of AA-DA (216 mV), DA-UA (170 mV) and UA-AA (386 mV) demonstrates that it is possible to determine AA, DA and UA at the DWGS-HCPC/GCE simultaneously using differential pulse voltammetry.

## 3. Materials and Methods

### 3.1. Chemicals

The discarded walnut (green) husks (Juglans regia epicarp) were obtained from the walnuts picked at a local walnut plantation (Xingtai, China). Uric acid (UA), acetaminophenol (APAP), glucose (GLU), ascorbic acid (AA) and dopamine (DA) were purchased from Shanghai Aladdin Biochemical Technology Co., Ltd. (Shanghai, China). Citric acid (CA) was purchased from Tianjin Yili Chemical Reagent Co., Ltd. (Tianjin, China). N,N-dimethylformamide (DMF) was provided by Guangfu Technology Development Co., Ltd. (Tianjin, China). The commercial beverage (water-soluble C100, produced by Nongfu Spring Co., Ltd., Zhejiang, China) was provided by the campus supermarket in Xingtai University (Xingtai, China).

### 3.2. Apparatus

Scanning electron microscopy (SEM) was performed using a Hitachi S-4800 ESEM (Hitachi Seisakusho Co., Ltd., Tokyo, Japan). X-ray diffraction (XRD) was carried out on Shimadzu XRD-6100 (Shimadzu, Tokyo, Japan). An ASAP 2460 system was used to conduct the nitrogen adsorption–desorption isotherm. The Raman spectroscopy analysis was characterized using the Renishaw Raman system model 1000 spectrometer (Gloucestershire, UK). The Fourier transform infrared (FT-IR) spectrum was obtained by the WQF-510A infrared spectrometer (Beijing Beifeng Rui Li Analytical Instruments (Group) Co., Ltd., Beijing, China). A freezer dryer (Chirst, Alpha 1–2 LD plus, MarinChrist Co., Ltd., Osterode, Germany) was used to perform the freeze-drying treatment. The electrochemical measurements were conducted using a CHI 660e electrochemical workstation (Chenhua Instrument Co., Ltd., Shanghai, China) employing a three-electrode configuration, where a platinum wire served as the counter electrode, the modified electrode functioned as the working electrode, and an Ag/AgCl electrode was utilized as the reference electrode. Unless otherwise specified, the electrolyte we selected for the electrochemical test was 0.1 M PBS with a pH of 7.0. According to the literature, the extremely alkaline pH values do not favor the redox process of ascorbic acid. Similarly, the oxidation of AA includes the transfer of two electrons and 2H^+^ ions, to produce dehydroascorbic acid, which was proven to be followed by an irreversible solvation reaction at pH lower than 4.0 [11,51]. This irreversible reaction yields an electroinactive product (2,3-diketogulonic acid) that is easily adsorbable on the electrode surface, which can result in electrode fouling [11,51]. Based on our current research, for the testing of real samples, we add a small amount of the real sample (soft drinks) to a neutral buffer solution for the detection.

### 3.3. Synthesis of the Three-Dimensional Honeycomb-like Porous Carbons Derived from Discarded Walnut (Green) Husks (DWGH-HCPCs)

Initially, walnut green husks were manually separated from walnuts and thoroughly washed with ultrapure water to remove surface impurities. Subsequently, the cleaned husks were cut into small segments and subjected to hydrothermal carbonization in a sealed autoclave maintained at 180 °C for 10 h. The processed material was rinsed with ultrapure water and subjected to 48 h of thermostatically controlled water bath treatment at 60 °C to remove water-soluble impurities. After cooling to ambient temperature, the purified material was stored in a refrigerator at −20 °C for preservation. Prior to pyrolysis, the frozen samples underwent lyophilization in a vacuum freeze-dryer for 48 h. The desiccated material was then transferred to ceramic boats and carbonized in a tube furnace under nitrogen atmosphere at 800 °C (2 h) with heating/cooling rates of 5 °C min^−1^. The resulting carbonized product (DWGS-HCPCs) was finally pulverized through intensive grinding to obtain the carbon powders.

### 3.4. Biosensors Preparation

The DWGS-HCPCs suspension (10 mg mL^−1^) was prepared by dispersing DWGS-HCPCs powder in DMF. A suspension of 8 µL of DWGS-HCPCs-DMF was dropped onto a bare glassy carbon electrode (GCE) (dia. 3 mm) and then dried under an infrared lamp. The resulting electrode was named DWGS-HCPC/GCE.

## 4. Conclusions

In this work, discarded walnut (green) husks, which constitute an abandoned biomass, are utilized as the raw material for the synthesis of DWGS-HCPCs, which presents the intriguing features of a porous structure and a large density of defective sites. The overpotential for AA electrooxidation may be clearly reduced by DWGS-HCPC/GCE, according to the comparison of the CVs at GCE and DWGS-HCPC/GCE in the presence of AA. In comparison with those at GCE, the manufactured amperometric sensor based on DWGS-HCPCs shows better analytical performance for AA oxidation with higher sensitivities (34.7 μA mΜ^−1^ and 22.7 μA mΜ^−1^), larger linear range (10–1040 μM and 1040–3380 μM) and lower detection limit (0.26 μM). Crucially, the DWGS-HCPC was able to measure AA in real samples with success and produce reliable results. The detection of AA at mM levels is analytically straightforward and can be achieved using low-tech methods. The approach in this work has the great potential of using DWGS-HCPCs as a relatively inexpensive and environmentally friendly nanomaterial in complex matrices, interference-prone environments, or a variety of applications (such as the food business and clinical diagnosis). Future work will expand to more challenging analytes such as dopamine and uric acid in biological fluids.

## Figures and Tables

**Table 1:** Electrochemical performance of different nanomaterial-based electrochemical AA sensors.

Sensors	Method	Linear Range (μM)	LOD (μM)	Samples	Reference
PPy-BPQDs-MIPs/PEDOTNRs/GCE ^1^	DPV	10–4000	3.3	Soft drinks	[44]
CA/MWCNTs/PVP/GCE ^2^	DPV	10–100	3	Chewable tables of vitamin C	[45]
MWCNT/poly-CD/GR/GCE ^3^	DPV	5–480	1.65	Human urine	[46]
Perylenetetracarboxylic acid/Gr/GCE ^4^	DPV	20–420	5.6	/	[47]
EPGrO/(Au/Ag/Pd)NPs/GCE ^5^	DPV	5–650	0.24	Human blood serum	[48]
MWCNT-polyArg/GCE ^6^	DPV	6–70	0.95	Bayaspirin C Effervescent	[49]
DWGS-HCPC/GCE	Amperometry	10–1040 1040–3380	0.26	Soft drinks	this work

^1^ Glassy carbon electrode/molecularly imprinted polymer (MIP) nanocomposites consisting of both conducting polypyrrole (PPy) and a new two-dimensional layered graphene-like black phosphorene quantum dots (BPQDs) onto the surface of conducting poly(3,4-ethylenedioxythiophene) nanorods (PEDOTNRs). ^2^ Glassy carbon electrode/nanofiber membranes of cellulose acetate (CA)/multi-walled carbon nanotubes (MWCNTs)/polyvinylpyrrolidone (PVP). ^3^ Glassy carbon electrode/multiwalled carbon nanotubes/polycyclodextrin. ^4^ Glassy carbon electrode/perylenetetracarboxylic acid/graphene. ^5^ Glassy carbon electrode/electropretreated graphene oxide/trimetallic nanoparticles (Au/Ag/Pd). ^6^ Glassy carbon electrode/multi-walled carbon nanotubes (MWCNT) with polyarginine (polyArg).

## Data Availability

All data are available in the manuscript.

## Supplementary Materials

The following supporting information can be downloaded at https://www.mdpi.com/article/10.3390/molecules30142980/s1, Figure S1: EIS curves at GCE and DWGS-HCPCs/GCE in 0.1 M KCl containing 5 mM Fe(CN)_6_^3−/4−^; Figure S2: Storage stability of the DWGS-HCPCs/GCE; Figure S3: Reproducibility of the DWGS-HCPCs/GCE; Table S1: Performance of AA detection with different methods.
