# Supplementary material for: A Sensitive and Accurate Electrochemical Sensor Based on Biomass-Derived Porous Carbon for the Detection of Ascorbic Acid
Source: Molecules. 2025 Jul 15;30(14):2980. doi: 10.3390/molecules30142980 (PMC12298935; doi:10.3390/molecules30142980)
Supplement: Supplementary file 1 [file molecules-30-02980-s001.zip › molecules-3716929-supplementary.pdf]

A sensitive and accurate electrochemical sensor based on biomass-derived porous carbon for the detection of Ascorbic Acid

Yashuang Hei [1,2,*], Lisi Ba [1,3], Xingwei Shi [2], Huanhuan Guo [1,3], Sisi Wen [1], Bingxiao Zheng [1,3], Wenjie Gu [3], and Zhiju Zhao [1,2,*]

1 Functional Polymer Materials Research and Development and Engineering Application Technology Innovation Center of Hebei Province, Xingtai University, Xingtai, Hebei Province 054001, China
2 Xingtai Key Laboratory of Biomimetic and Catalytic Materials, Xingtai, Hebei Province 050041,China
3 College of Chemical Engineering and Biotechnology, Xingtai University, Xingtai, Hebei Province 054001, China

* Correspondence: heiys0815@163.com; zhaozj2005@163.com

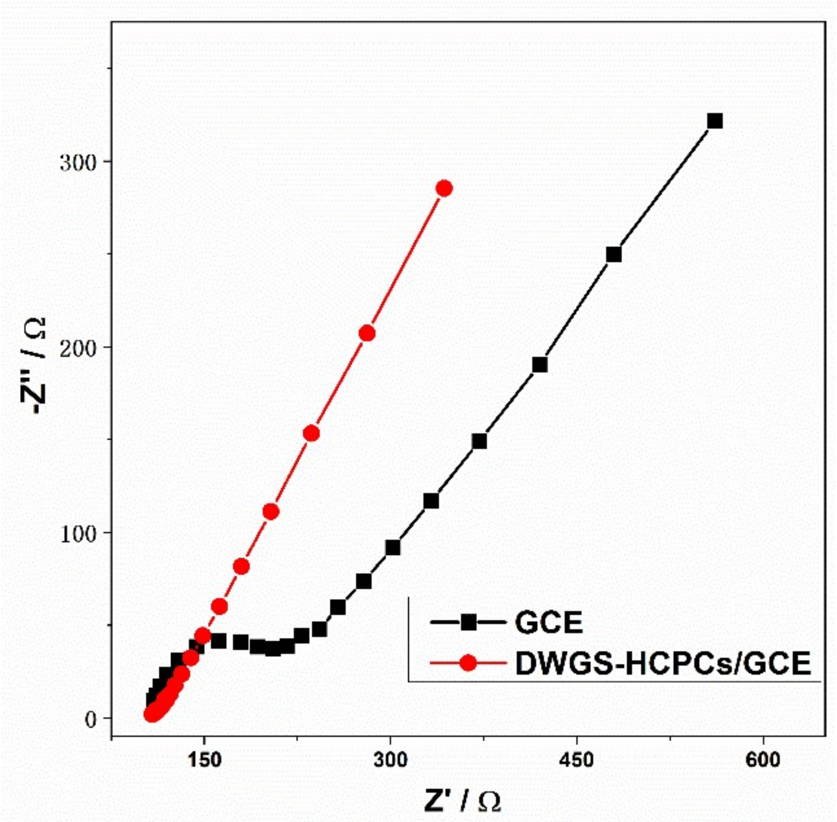

Figure S1. EIS curves at GCE and DWGS-HCPCs/GCE in 0.1 M KCl containing 5 mM $Fe(CN)_6^{3-/4-}$.

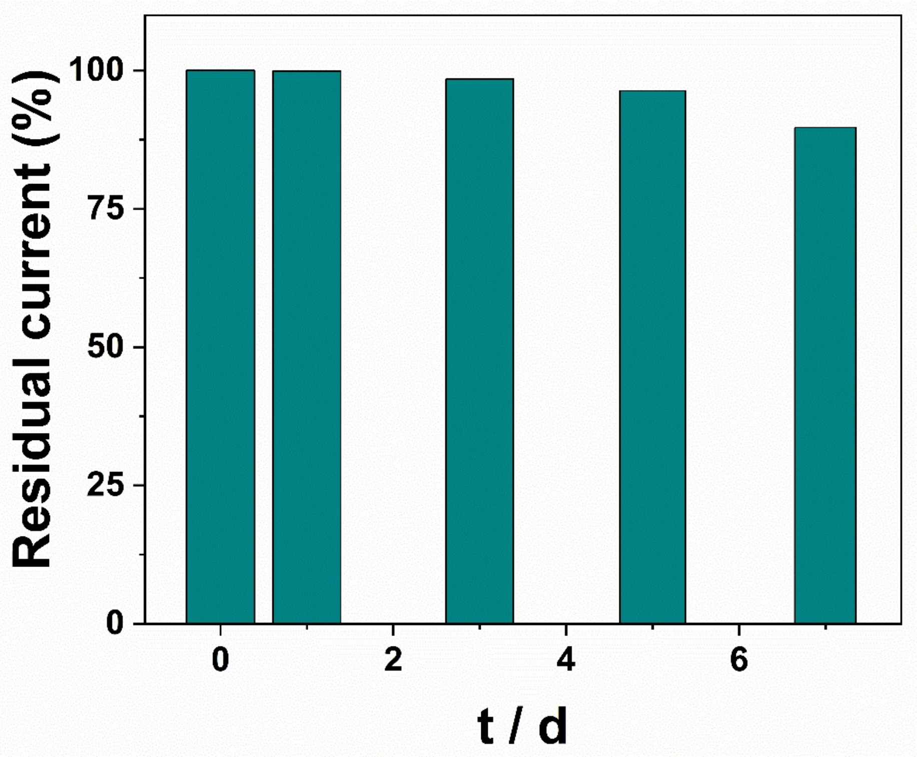

Figure S2. Storage stability of the DWGS-HCPCs/GCE.

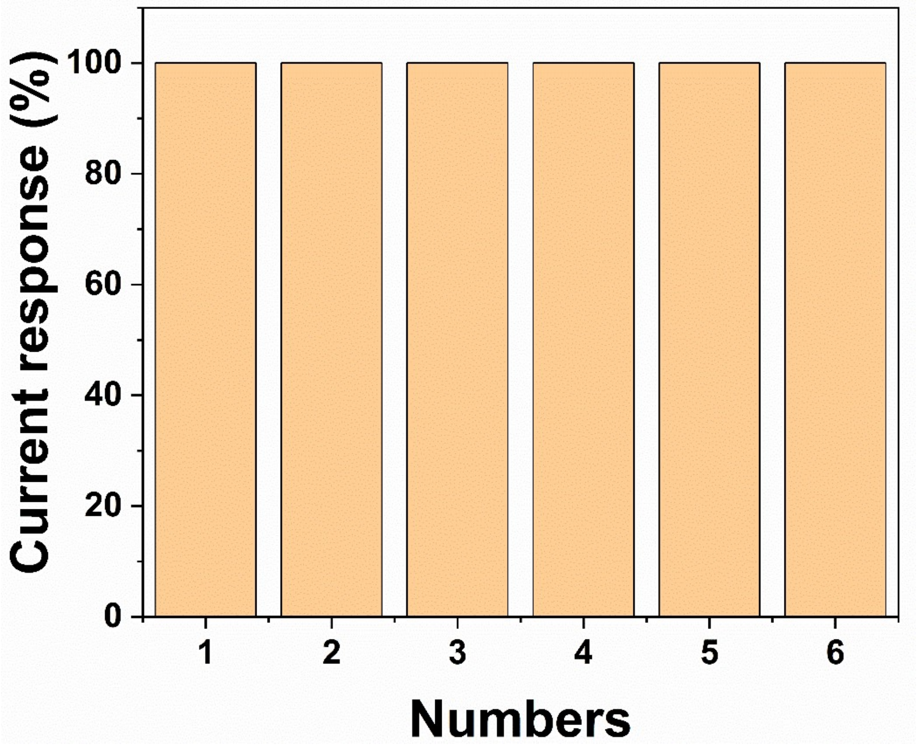

Figure S3. Reproducibility of the DWGS-HCPCs/GCE.

Table S1. Performance of AA detection with different methods.

Materials	Method	Linear range (μM)	LOD (μM)	Samples	Reference
Protein-stabilized gold nanoclusters	Fluorescence analyses	0.1-10	0.022	fruit and beverage samples	[13]
/	High-performance liquid chromatography	10^{-5}–0.01	1.09×10^{-13}	cellular extracts	[14]
/	Capillary electrophoresis	14.2-283.9	/	fruit juice	[15]
/	Spectrophotometric method	56.8-1135.7	11.4		[16]
Au@MnO_2 NPs	UV–vis spectrum	0.75-17.5	0.18/0.47	vitamin C tablets	[51]
DWGS-HCPCs/GCE	Amperometry	10-1040<br>1040-3380	0.26	Soft drinks	this work
